# Consensus Guidelines on Genetic` Testing for Hereditary Breast Cancer from the American Society of Breast Surgeons

**DOI:** 10.1245/s10434-019-07549-8

**Published:** 2019-07-24

**Authors:** Eric R. Manahan, Henry M. Kuerer, Molly Sebastian, Kevin S. Hughes, Judy C. Boughey, David M. Euhus, Susan K. Boolbol, Walton A. Taylor

**Affiliations:** 1 Department of Surgery, Hamilton Medical Center, Dalton, GA USA; 2 Department Breast Surgical Oncology, MD Anderson Cancer Center, Houston, TX USA; 3 Reinsch Pierce Family Center for Breast Health, Virginia Hospital Center, Arlington, VA USA; 4 Department of Surgical Oncology, Massachusetts General Hospital, Boston, MA USA; 5 Department of Surgery, Mayo Clinic, Rochester, MN USA; 6 Department of Surgery, Johns Hopkins Hospital, Baltimore, MD USA; 7 Department of Surgery, Mount Sinai Beth Israel, New York, NY USA; 8 Texas Health Physicians Group, Dallas, TX USA

## Abstract

**Background:**

The purpose of this consensus guideline is to outline recommendations for genetic testing that medical professionals can use to assess hereditary risk for breast cancer.

**Methods:**

Literature review included large datasets, basic and clinical science publications, and recent updated national guidelines. Genetic testing to assess hereditary risk of cancer is a complex, broad, and dynamic area of medical research. The dominant focus of this guideline is limited in scope to breast cancer.

**Results:**

There is a lack of consensus among experts regarding which genes among many should be tested in different clinical scenarios. There is also variation in the degree of consensus regarding the understanding of risk and appropriate clinical management of mutations in many genes.

**Conclusions:**

Genetic testing should be made available to all patients with a personal history of breast cancer. Recent data are reviewed that support genetic testing being offered to each patient with breast cancer (newly diagnosed or with a personal history). If genetic testing is performed, such testing should include BRCA1/BRCA2 and PALB2, with other genes as appropriate for the clinical scenario and family history. For patients with newly diagnosed breast cancer, identification of a mutation may impact local treatment recommendations. Patients who had genetic testing previously may benefit from updated testing. Genetic testing should be made available to patients without a history of breast cancer who meet National Comprehensive Cancer Network guidelines. Finally, variants of uncertain significance are not clinically actionable and these patients should be managed based on their individual risk factors.

The American Society of Breast Surgeons recently reviewed the use of genetic testing for patients with breast cancer. An expert panel from a variety of backgrounds reviewed the current literature related to genetic testing and produced an updated consensus statement that the board of directors approved. This is now the official updated position statement of the American Society of Breast Surgeons (Table 1). Our leadership concluded that we must change our official recommendations for genetic testing such that genetic testing should be made available to all interested patients diagnosed with breast cancer.

**Table 1:** Overall recommendations for genetic testing for hereditary breast cancer from the American Society of Breast Surgeons

*Breast surgeons, genetic counselors, and other medical professionals knowledgeable in genetic testing can provide patient education and counseling and make recommendations to their patients regarding genetic testing and arrange testing*
When the patient’s history and/or test results are complex, referral to a certified genetic counselor or genetics professional may be useful. Genetic testing is increasingly provided through multigene panels. There are a wide variety of panels available, with different genes on different panels. There is a lack of consensus among experts regarding which genes should be tested in different clinical scenarios. There is also variation in the degree of consensus regarding the understanding of risk and appropriate clinical management of mutations in some genes
*Genetic testing should be made available to all patients with a personal history of breast cancer*
Recent data support that genetic testing should be offered to each patient with breast cancer (newly diagnosed or with a personal history). If genetic testing is performed, such testing should include BRCA1/BRCA2 and PALB2, with other genes as appropriate for the clinical scenario and family history. For patients with newly diagnosed breast cancer, identification of a mutation may impact local treatment recommendations (surgery and potentially radiation) and systemic therapy. Additionally, family members may subsequently be offered testing and tailored risk reduction strategies
*Patients who had genetic testing previously may benefit from updated testing*
Every patient being seen by a breast surgeon, who had genetic testing in the past and no pathogenic variant was identified, should be re-evaluated and updated testing considered. In particular, a patient who had negative germline BRCA1 and 2 testing, who is from a family without pathogenic variants, should be considered for additional testing.^1^ Genetic testing performed prior to 2014 most likely would not have had PALB2 or other potentially relevant genes included and may not have included testing for large genomic rearrangements in BRCA1 or BRCA2
*Genetic testing should be made available to patients without a history of breast cancer who meet NCCN guidelines*
Unaffected patients should be informed that testing an affected relative first, whenever possible, is more informative than undergoing testing themselves. When it is not feasible to test the affected relative first, then the unaffected family member should be considered for testing if they are interested, with careful pre-test counseling to explain the limited value of “uninformative negative” results. It is also reasonable to order a multi-gene panel if the family history is incomplete (i.e., a case of adoption, patient is uncertain of exact type of cancer affecting family members, among others) or other cancers are found in the family history, as described above
*Variants of uncertain significance are DNA sequences that are NOT clinically actionable*
This type of result needs to be considered as inconclusive, and the patient should be managed based on their risk factors and not influenced by this result

National guidelines were originally established to help identify patients who had a high likelihood of benefiting from genetic testing that looked only for *BRCA 1/2* mutations. The initial threshold for testing was set high because at that time genetic testing was very expensive and was just beginning to be used for medical care. The cost of testing has dropped dramatically (panel genetic testing can cost less than a diagnostic mammogram with an ultrasound), and the benefit to the patient and the patient’s family can be lifesaving. Unfortunately, we still see evidence that the guidelines deny patients’ access to this important testing and the valuable information it provides. Put simply, the guidelines have become more about exclusion than inclusion. This consensus statement reviews the available literature although not an exhausted systematic review, a comprehensive review of the most impactful evidence in modern literature on the subject. These guidelines now supersede similar guidelines from our society put forward in 2006, 2012, 2016, and 2017. Based on the most compelling available data to review, five clearly articulated recommendations are made for members of our society and patients with breast cancer.

## Summary of Data Reviewed

The National Cancer Institute estimates for 2018 were that more than 266,000 new cases of invasive breast cancer would be diagnosed in the United States, and more than 40,000 patients would die from the disease.^2^ Approximately 10% of breast cancers are associated with a pathogenic germline variant in one of several different genes.^3^ More than 50% of pathogenic germline variants are mutations in the *BRCA1* and *BRCA2* genes.^4^–^9^ Using genetic testing to identify patients who are at increased risk to develop breast cancer enables patients to take steps to reduce this risk. There are several risk management strategies available for individuals at increased risk (e.g., chemoprevention along with enhanced screening; risk reducing surgeries).^10^–^18^ Unfortunately, in the current state of medical practice, a significant number of pathogenic mutation carriers remain undetected and undiagnosed. These are largely women with “moderate penetrance” mutations, but even women with *BRCA1 or 2* mutations may not be identified.^19^–^22^ There is an unmet challenge to improve our identification and diagnosis of patients who have an inherited increased lifetime risk of breast cancer.

### Access to Genetic Counseling and Testing

There are fewer barriers to genetic testing now than previously, and testing is less costly and being offered by more labs. The indications for who should be offered testing are ever increasing—each guideline update casting a wider net, and there is more public awareness. However, some barriers remain—one of which is the limited availability of genetic counseling nationwide for patients and their family members.^19^–^22^

Increased access to testing would likely lead to more patients pursuing testing and improving rates of identification of gene carriers. Breast surgeons are well positioned to be a resource for patients who may benefit from testing. Breast surgeons can identify individuals who are suitable for testing, inform patients of the risks and benefits, provide access to genetic testing, and also discuss risk management strategies for those patients who test positive. For patients with less common mutations, strong consideration should be given to consultation with cancer genetics specialists.^23^–^25^

### Hereditary Breast Cancer Syndromes

Hereditary mutations to be considered include *BRCA 1&2, PALB2*, and other hereditary breast cancer syndromes, which include but are not limited to Li-Fraumeni syndrome (*TP53* pathogenic variant), Cowden syndrome (*PTEN* pathogenic variant), hereditary diffuse gastric cancer syndrome (*CDH1* pathogenic variant), and Peutz-Jegher syndrome (*STK11* pathogenic variant).

### Impact of Genetic Testing Results on Management Recommendations

Identification of patients with pathogenic variants in these genes can influence patient management in terms of high-risk screening and risk reduction as well as therapeutic options related to surgery, radiation, and systemic therapies.^26^–^28^ For example, identifying that a breast cancer patient has a *BRCA1* pathogenic variant provides that patient the opportunity to learn of her elevated risk for contralateral breast cancer as well as of ovarian cancer and to make educated decisions to reduce those risks.^28^ Studies are underway to determine whether these patients also might benefit from PARP inhibitors being included in their adjuvant therapy regimen. Another example is that radiation is relatively contraindicated in patients with *TP53* pathogenic variants (associated with Li-Fraumeni Syndrome) due to their increased risk of developing radiation-induced secondary malignancies.

Identifying a patient who has a pathogenic variant that indicates high hereditary breast cancer risk can have a profound impact on that patient’s health and management. Additionally, it has potential impact on that patient’s family members who should be counselled to consider testing for the mutation identified in the family, the result of which can guide their risk of breast cancer development and consideration of risk management strategies.

Just because a hereditary pathogenic mutation that predisposes to breast cancer is identified does not mean that the risk-reducing mastectomy is indicated. Risk-reducing mastectomy can be considered in *BRCA1, BRCA 2, PTEN, and TP53*. Consideration may also be appropriate for patients with mutations in other genes when combined with a significant family history of breast cancer.

Patients with *BRCA1 or BRCA2* pathogenic variants should consider risk-reducing bilateral salpingo-oophorectomy after child-bearing or between the ages of 35–40 years to reduce ovarian and fallopian tube cancer risk. Women with *BRCA1* should consider oophorectomy between ages 35–40 years, whereas *BRCA2* carriers should consider it between ages 40–45 years.

Prophylactic oophorectomy in premenopausal women with *BRCA2* pathogenic variants also has been shown to reduce the risk of breast cancer by approximately 50%. There also is breast cancer risk reduction from RRSO in *BRCA1* patients but to a lesser degree.^10^,^11^,^17^

For patients with mutations in *ATM, CDH1, CHEK2, NBN, NF1, PALB2, and STK11*, enhanced screening is recommended; however, currently the data are not sufficient to support risk-reducing mastectomy in the absence of other factors such as a strong family history. There are substantial gaps in our ability to predict individual risks associated with mutations in some of these genes. Risk is modulated by age, family history, and in some cases, the specific mutation in a particular gene. For the aforementioned syndromes, the guidelines broadly support considering mammography with tomosynthesis and breast MRI with and without contrast for annual screening due to the elevated risk for breast cancer.

For *BARD1, MSH2, MLH1, MSH6, PMS2, EPCAM, BRIP1, RAD51C,* and *RAD51D*, there are some data, suggesting an elevated lifetime risk of breast cancer; however, there is insufficient evidence to support change in breast cancer risk management based on the presence of a mutation alone. Mutations in these genes may be associated with an increased risk of gynecological cancers, which may warrant specific management. *MSH2, MLH1, MSH6*, and *PMS2* are associated with the Lynch Syndrome, a multiorgan predisposition syndrome that requires multidisciplinary management.

The list of actionable genes and recommendations for screening and risk management continually evolves as additional information becomes available. We refer the readers to the NCCN guidelines, available online at www.nccn.org under the title Familial High-Risk Assessment: Breast and Ovarian Cancer (most recently updated in early 2019). The All Syndromes Known to Man Evaluator (https://ask2me.org/) is another tool available with information on the spectrum and estimated penetrance for pathologic variants.^29^

### Limitations of Genetic Testing

Health care providers and patients need to know that genetic testing is one of several tools for assessing breast cancer risk. Not every genetic test yields a straightforward answer with clear guidance on how to proceed for optimal care. Patients should be made aware that negative test results do not necessarily mean they are not at increased risk for developing breast cancer.

Many factors contribute to a patient’s lifetime risk of breast cancer, and genetic testing is an effort to better define one of these elements (the measurable inherited risk). When counseling patients about their lifetime risk of breast cancer, it is critical to look at the patients’ other contributing factors, such as age, medical history, lifestyle, exposures, and family history. For patients who test positive for a pathogenic variant, it is important to gain detailed understanding of that variant when advising on risk management strategies—details, such as the penetrance of the cancer risk among carriers (how likely is the patient to actually develop breast cancer). Penetrance varies among the identified hereditary cancer syndromes. Not all carriers of pathogenic genetic variants will develop breast cancer, and the level of risk varies with the gene affected and likely the variant as well.^6^,^30^,^31^ Some types of *CHEK2 and ATM* variants have low penetrance, whereas other types are more highly penetrant.^32^,^33^ Ask2me.org can be useful in understanding the penetrance and the management for most cancer-causing genes, and the BRCA Decision Tool, http://brcatool.stanford.edu/brca.html, can be useful in known BRCA pathogenic variant carriers to predict likelihood of developing breast or ovarian cancer and likelihood of dying from either disease based on patient age and a variety of interventions chosen for screening and prophylaxis. It is important to note that these calculators are constrained by the limitations of the studies that provide the underlying odds ratios used to generate the absolute risk estimates and do not account for modification of those odds ratios by age, mutation position, family history, or polygenic background risk.^34^

### Pre-and Post-test Counseling

Before testing, patients need to be made aware of the implications that the test result can have (pre-test counseling); and when results become available, patients should be reminded of these implications and be provided the appropriate clinical context for the results to make informed decisions (post-test counseling). All genetic testing should be performed in the setting of informed consent. The American College of Surgeons Commission on Cancer accreditation program mandates that cancer risk assessment, counseling, and genetic testing services be provided to patients by a physician who does risk assessment regularly and/or is qualified to do testing or a qualified genetic professional either on site or by referral.^35^ A systematic review of the literature indicates that pre-test counseling, whether by a geneticist, breast surgeon, oncology nurse, or other medical professional with expertise and experience in cancer genetics reduces distress, improves risk perception accuracy, and improves follow through for testing.^36^ Breast surgeons who are knowledgeable in cancer genetics can initiate and guide genetic testing for their patients. Pre-test counseling should include discussion of the types of results (true positive = pathogenic, true negative = benign (although without a known positive in a family, it also may be inconclusive as well), and inconclusive = variant of uncertain significance (VUS)). Other potential issues of testing should be reviewed, such as inconclusive results, misperception of true risk, and discrimination. As noted above, patients need to know there are limitations to this testing including noninformative results or negative tests as well as the reality of the evolving science. It is important to educate patients on the benefits of testing as a vehicle to knowing better their individual risk and empowerment to consider interventions to manage or reduce that risk. It can be helpful to set expectations for when the test results will be available.

Post-test counseling is important regardless of the actual result. The current best practice is for all patients who undergo genetic testing to have some form of post-test counseling. By NCCN guidelines, this can occur in person or remotely. This allows for patients’ questions to be answered and for a thorough debriefing. If a result is negative or noninformative (such as a variant of uncertain significance [VUS]), then the patient’s other risk factors for breast cancer (age, medical history, family history, etc.) need to be evaluated to formulate the appropriate risk management plan. Depending on the level of risk for breast cancer, strategies to manage that risk can be discussed, including enhanced screening imaging (annual mammogram and breast MRI); chemoprevention (endocrine therapy to lower risk); lifestyle modification with respect to obesity, tobacco use, and alcohol consumption; and exogenous hormone use among others.

For patients who test positive for a pathogenic variant, a clear review of the state of evidence for that specific syndrome is imperative. To make educated decisions, patients need to know about the spectrum of risk management strategies. Ultimately, a customized plan for the patient is the goal with their informed consent. In this discussion, a frank statement of the level of risk reduction for each intervention is needed. For example, risk-reducing mastectomy and reconstruction in a BRCA1-positive 35-year-old patient leads to much greater risk reduction for breast cancer mortality than that same intervention in a 65-year-old patient.^23^,^37^,^38^ The surgeon should discuss these issues and refer to other specialists (such as gynecologic oncologists, gastroenterologists, etc.) for other organs at risk as appropriate. For complex scenarios, referral to a genetics professional is recommended.

### Multi-Gene Panel Testing

Genetic testing has expanded in scope and availability since 2013 when the U.S. Supreme Court ruling in Association for Molecular Pathology v. Myriad Genetics, Inc. increased the testing options. Increased competition has helped to lower the cost. Improvements in technology, such as next-generation sequencing, has made testing for more than one gene at a time a reality, which can improve the cost-effectiveness and efficiency of testing.^39^–^43^ While *BRCA1 and BRCA2* remain the most likely genes to be mutated in a family with high breast and ovarian cancer risk, panel testing can allow for more comprehensive coverage of less common syndromes that can also confer hereditary cancer risk.^4^,^7^,^23^,^44^–^47^ Numerous recent studies have shown that panel testing can significantly increase the rate of detection of pathogenic variants, with the most frequently identified pathogenic variants (outside of *BRCA1 and BRCA2*) being in *PALB2, CHEK2, and ATM*.^4^,^23^,^46^ As previously noted, there is a comparatively limited understanding of individual breast cancer risk associated with mutations in genes other than *BRCA1 and BRCA2*. However, the presence of mutations in *PALB2, ATM*, truncating mutations in *CHEK2*, and possibly other genes are likely to be associated with lifetime breast cancer risks of greater than 20% and therefore, in the United States, at least support a decision for enhanced surveillance with annual mammography with tomosynthesis and breast MRI with contrast. Mutations in other genes also may reach this threshold, although the rarity of such mutations and the possibility of subtype-specific predisposition make risk estimation more challenging. A multigene panel may include genes with varying degrees of evidentiary support and “actionability.” This testing method is optimal when the individual genes included are clinically valid and comprehensively address the details of each patient’s case.

Panel testing can be considered for patients who qualify for hereditary breast cancer testing to more efficiently and cost-effectively evaluate genes that confer risk and impact management recommendations. When genetic testing is being recommended based on phenotypic syndromes (e.g., 3 or more close family members affected by breast cancer at any age), then multigene panel testing is likely to be more efficient in evaluating patients. In fact, the most recent NCCN guidelines allow that panel testing will largely replace sequential gene sequencing (i.e., the older approach of evaluating *BRCA* pathogenic variants first, then selecting additional genes if *BRCA* tests are negative).20,30,43 Surgeons, genetic counselors, and other health care professionals who order panel testing for breast cancer patients or their family members should at a minimum test the breast cancer genes that are clinically actionable given the current state of medical evidence. Testing of additional genes can be performed at the discretion of the ordering physician or as directed by the family history.

### Variant of Uncertain Significance (VUS)

Variants of uncertain significance are DNA sequences that are NOT clinically actionable. This type of result needs to be considered as inconclusive. For example, a patient who receives a genetic testing result of “BRCA1 variant of uncertain significance” should NOT be recommended for a change in management based on that test result alone. No clinical treatment plan or risk management plan should be influenced by a VUS. These are DNA sequences about which the lab is still accruing data for definitive classification as to benign or pathogenic. The vast majority are re-classified as benign when enough data are collected. Usually, it takes several years for the reclassification to take place.^44^,^48^

The American College of Medical Genetics has published guidelines for reporting DNA sequence variations.^49^ The rate of identifying VUSs can be high when new syndromes are identified but that rate decreases as data regarding those genes and the VUSs are accrued. Current rates of identifying a VUS with newer multigene panel testing is reported to be between 6.7 and 41.7%.^23^,^44^–^46^ There are still VUSs identified with BRCA1/2 testing. However, the rates are generally much lower, ranging from 2 to 5%, now that testing of these two syndromes has been available for more than 20 years. In general, patients with VUSs should be managed based on their family history, medical history, age, and other factors that influence breast cancer risk. No weight should be given to the VUS found, and co-segregation among affected family members is not conclusive evidence of pathogenicity.

## Recommendations and Conclusions

Table 1 summarizes the overall recommendations on genetic testing for hereditary breast cancer. Members of the American Society of Breast Surgeons do not directly manage all the genetic disorders that may now be identified in testing. However, we advocate multidisciplinary, team-based patient management because our members are well positioned to do this, and they should, for patients’ benefit, work with multiple specialties that can identify and manage these findings effectively.

While surgeons unfamiliar with hereditary cancer syndromes should not interpret the results, the number of breast surgeons trained to do this interpretation is increasing rapidly due to its growing importance in day-to-day care and our society’s concerted efforts to educate our membership. Breast surgeons understand or can learn that managing a mutation varies based on many factors, that a negative genetic testing result is of little value without proper context, and that a variant of uncertain significance is not clinically significant, among other nuances. The ASBrS provides courses at every annual meeting that cover when to order testing on affected and nonaffected patients, how to manage the results, and how to conduct proper counseling of patients and their families. Online resources such as Ask2Me.Org (http://ask2me.org), ClinVar (https://www.ncbi.nlm.nih.gov/clinvar/), and NCCN (https://www.nccn.org) are available to help surgeons and other providers manage patients with hereditary conditions.

As genetic testing expands, it is possible that laboratories with inadequate quality standards will appear in the marketplace. It is important to choose the lab carefully making sure they provide quality testing with accurate results and appropriate follow-up.

These are guidelines, not rules, and there are patients for whom these guidelines will not apply. However, too many patients develop cancers that might have been prevented or found earlier if genetic testing had been performed. Our society has a responsibility to offer genetic testing to those interested patients in order to act when we see an opportunity to decrease unnecessary morbidity and mortality. We do so today with the adoption of our new position on this issue.
